# Platelet Rich Fibrin (PRF) and Its Related Products: Biomolecular Characterization of the Liquid Fibrinogen

**DOI:** 10.3390/jcm9041099

**Published:** 2020-04-12

**Authors:** Giorgio Serafini, Mariangela Lopreiato, Marco Lollobrigida, Luca Lamazza, Giulia Mazzucchi, Lorenzo Fortunato, Alessia Mariano, Anna Scotto d’Abusco, Mario Fontana, Alberto De Biase

**Affiliations:** 1 Department of Oral and Maxillo Facial Sciences, “Sapienza” University of Rome, Via Caserta 6, 00161 Rome, Italy; 2 Department of Biochemical Sciences “Alessandro Rossi Fanelli”, “Sapienza” University of Rome, Piazzale Aldo Moro 5, 00185 Rome, Italy

**Keywords:** liquid fibrinogen, platelet concentrate, growth factors, platelet rich fibrin, PRF, platelets, leukocyte

## Abstract

Liquid fibrinogen is an injectable platelet concentrate rich in platelets, leukocytes, and fibrinogen obtained by blood centrifugation. The aim of this study was to analyze the release of different growth factors in the liquid fibrinogen at different times and to assess possible correlations between growth factors and cell counts. The concentration of transforming growth factor beta 1 (TGF-β1), platelet-derived growth factor-AB (PDGF-AB), platelet-derived growth factor-BB (PDGF-BB), bone morphogenetic protein 2 (BMP-2), fibroblast growth factor 2 (FGF-2) and vascular endothelial growth factor (VEGF) released by liquid fibrinogen were examined with ELISA at three time points (T0, time of collection; T7, 7 days; T14, 14 days). The cellular content of the liquid fibrinogen and whole blood was also calculated for each volunteer. A mean accumulation of platelets of almost 1.5-fold in liquid fibrinogen compared to whole blood samples was found. An increase of TGF-β1, PDGF-AB, FGF-2, and VEGF levels was detected at T7. At T14, the level of TGF-β1 returned to T0 level; PDGF-AB amount remained high; the levels of FGF-2 and VEGF decreased with respect to T7, but remained higher than the T0 levels; PDGF-BB was high at all time points; BMP-2 level was low and remained constant at all time points. TGF-β1, PDGF-AB, and PDGF-BB showed a correlation with platelet amount, whereas BMP-2, FGF-2, and VEGF showed a mild correlation with platelet amount. Due to the high concentration of platelets, liquid fibrinogen does contain important growth factors for the regeneration of both soft and hard tissue. The centrifugation protocol tested in this study provides a valid solution to stimulate wound healing in oral and periodontal surgery.

## 1. Introduction

The focus of regenerative medicine is the restoration of damaged tissues [1]. A stable fibrin clot is the first step of the healing process as it provides a provisional matrix for the migration of mesenchymal cells, fibroblasts, and epithelial cells. This has been the rationale for using platelet gel and fibrin glues in oral and maxillofacial surgery [2,3].

Research and development of new protocols to enhance hemostasis and wound healing is, however, a common focus for all surgical disciplines. The interest for innovative techniques has been particularly evident in the development of autologous platelets concentrates for surgical use, given their high concentration of fibrin [4]. For many years, platelet concentrates have been used as surgical adjuvants in oral and maxillofacial surgery, sports medicine, orthopedic surgery, and esthetic plastic surgery [5,6,7,8,9]. To improve tissue healing and regeneration, platelet concentrates are also used to obtain a local release of growth factors [10].

Focusing on oral surgery, platelet concentrates have been proven to promote and stimulate wound healing processes and to accelerate angiogenesis. Del Fabbro et al. [11] confirmed the use of growth factors for providing an additional stimulation to physiological healing processes. Specifically, improved hemostasis, greater protection of the post-extraction alveolus, reduction in post-operative pain, and more rapid epithelialization have been highlighted.

Platelet concentrates are classified as platelet-rich plasma (PRP) or platelet-rich fibrin (PRF). In PRP techniques, blood is collected with anticoagulant and then centrifuged, whereas in PRF techniques, blood is collected without any anticoagulant and immediately centrifuged [12].

As for leukocyte- and platelet-rich fibrin (L-PRF) products, including L-PRF clots and liquid fibrinogen, platelet concentrates are obtained by centrifugation of a whole blood sample, discarding red blood cells and concentrating the components to be used, such as fibrin, platelets, growth factors, leukocytes, and other circulating cytokines and proteins [13]. By pressing L-PRF clots, an exudate, rich in plasma proteins such as fibronectin, vitronectin, and thrombospondin-1 can be also obtained [5,14,15]. These plasma proteins have an important role in cell adhesion and migration into the fibrin clot and can further improve the early stages of wound healing [16].

Compared to L-PRF clots, a liquid concentrate of platelets, leukocytes, plasma proteins, and fibrinogen can be produced by shorter blood centrifugation [17]. This product, called liquid fibrinogen, is collected before coagulation and may be used for local delivery of growth factors similar to fibrin clots. However, compared to L-PRF clots, liquid fibrinogen has been less studied and characterized with respect to the amount of different growth factors. However, as demonstrated in a previous study [18], it may have potential applications for the biofunctionalization of dental implants.

The aim of this in vitro study was to analyze the release of different growth factors present in liquid fibrinogen at different times and to assess correlations between growth factors release and liquid fibrinogen cell counts.

## 2. Materials and Methods

### 2.1. Patient Selection

All procedures performed in this study were conducted in accordance with the Declaration of Helsinki of 1975, revised in 2013. All volunteers were treated according to a specific protocol approved by the Ethics Committee for Human Research of Sapienza University (date of approval: 7 November 2019; approval number: 906/19). A written informed consent was obtained from each participant before the treatment.

Ten systemically healthy volunteers (4 men and 6 women), aged from 40 to 50 years, were enrolled in this study. The exclusion criteria were anticoagulant and antiplatelet medication therapy, blood disorders, bone metabolism disorders, antiresorptive therapy (as bisphosphonates), pregnancy, or lactation.

### 2.2. Preparation of Blood Samples and Liquid Fibrinogen

**Figure 1:**
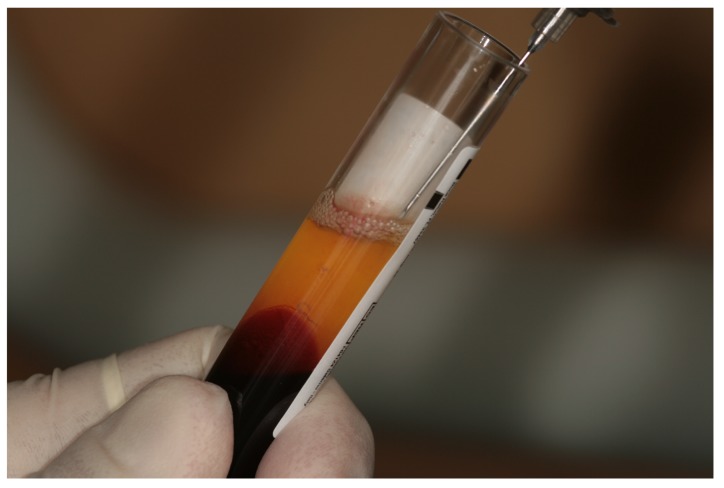
Liquid fibrinogen collection from white-cap tube.

Three samples of 9 mL of peripheral venous blood were collected from each volunteer for the biochemical analysis. One sample was collected in a blood count test tube with potassium EDTA as anticoagulant (BD Vacutainer^®^, Franklin Lakes, NJ, USA) to perform the complete blood count (CBC) test, and two samples were collected in noncoated tubes without anticoagulants (white-cap tubes, Intra-Lock, Florida, USA) to obtain liquid fibrinogen. The white-cap tubes were centrifuged at 2700 rpm for 3 min (RCF_clot_ = 408 *g*; RCF_max_ = 653 *g*; RCF_min_ = 326 *g*) [19] using an Intraspin™ centrifugation device (33° rotor angulation, 50 mm radius at the middle of the tube, 80 mm at the maximum, and 40 mm at the minimum) (Intra-Lock, Boca Raton, FL, USA), according to the manufacturer’s instructions. Immediately after centrifugation, the upper yellow fluid (liquid fibrinogen) in the white-cap tubes was recovered by sterile syringes, avoiding red blood cells, drawn into tubes containing potassium EDTA and processed for analyses (Figure 1). Of these last two samples, one was for CBC, the other for the analysis of the growth factors. The CBC tests were performed using a hematology analyzer (Advia 2120 Hematology System, Siemens Healthcare GmbH, Erlangen, Germany).

### 2.3. ELISA Analyses of Growth Factors

The amount of transforming growth factor beta 1 (TGF-β1), platelet-derived growth factor-AB (PDGF-AB), platelet-derived growth factor-BB (PDGF-BB), bone morphogenetic protein 2 (BMP-2), fibroblast growth factor 2 (FGF-2) and vascular endothelial growth factor (VEGF) in the liquid fibrinogen of each volunteer was determined using Enzyme-Linked Immunoadsorbent assay kits (Fine Test ELISA, Fine Biotech Co., Ltd., Wuhan, China) according to the manufacturer’s instructions. Optical Density (O.D.) absorbance was measured at 450 nm by a microplate reader (Appliskan, Thermo Fisher, Waltham, MA, USA). The samples were analyzed at the moment of collection, T0; at seven days after collection, T7; and fourteen days after collection, T14. The samples analyzed at T7 and T14 were left on the bench at room temperature.

### 2.4. Statistical Analysis

Each data point, within any single experiment, is the mean (± SD) of three independent replicas. Data were statistically analyzed with two-way repeated measures analysis of variance (ANOVA) with Bonferroni’s multiple comparison test, using Prism 6.0 software (GraphPad Software, San Diego, CA, USA).

## 3. Results

### 3.1. Complete Blood Count Test on Whole Blood and Liquid Fibrinogen

**Figure 2:**
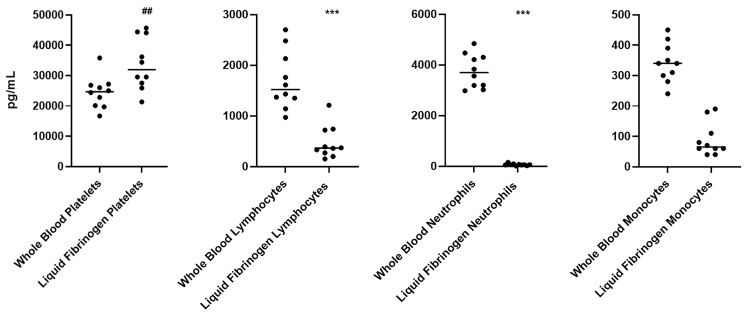
Cell count test on volunteers’ whole blood and liquid fibrinogen. The accumulation of platelets, lymphocytes, neutrophils, and monocytes is reported. Each data point, within any single experiment, is the mean (± SD) of three independent replicas. ## *p* < 0.01; *** *p* < 0.001.

At the time of collection, a complete blood count for all volunteers was carried out. Red blood cells, white blood cells, and platelets were counted in both whole blood and liquid fibrinogen, finding a statistically significant mean accumulation of platelets of almost 1.5-fold in liquid fibrinogen compared to whole blood samples (*p* < 0.01). No accumulation of all other blood cells was found in the liquid fibrinogen, rather a statistically significant decrease was observed for lymphocytes and neutrophils (*p* < 0.001) and a not statistically significant decrease of monocytes compared to whole blood samples (Figure 2).

### 3.2. Growth Factor Release in Liquid Fibrinogen

The release over time of TGF-β1, PDGF-AB, PDGF-BB, BMP-2, FGF-2 and VEGF in liquid fibrinogen from each donor was detected at three time points, respectively, at the time of liquid fibrinogen collection (T0), 7 days after collection (T7), and 14 days after collection (T14). An increase of TGF-β1, PDGF-AB, FGF-2, and VEGF was detected at T7, whereas at T14, the level of TGF-β1 returned to the T0 level; PDGF-AB amount remained high; the levels of FGF-2 and VEGF decreased with respect to T7, but remained higher than the T0 levels. PDGF-BB was high at all time points analyzed even if an increase at T14 was detected. BMP-2 level was low and remained constant at all time points analyzed.

**Figure 3:**
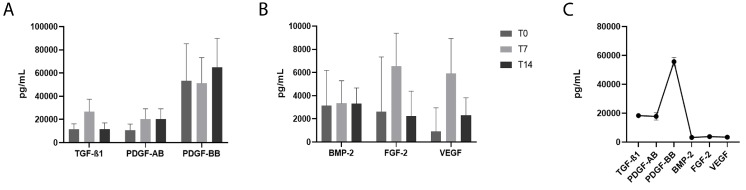
Growth factors’ release by liquid fibrinogen. The release of growth factors TGF-β, PDGF-AB, and PDGF-BB in (**A**), BMP-2, FGF-2, and VEGF in (**B**), was measured by ELISA at the moment of sample collection (T0), 7 days after collection (T7), and 14 days after collection (T14). The cumulative concentration of each factor, considering the 10 samples, calculated at T14 is reported in (**C**). TGF-β1, transforming growth factor beta 1; PDGF-AB, platelet-derived growth factor-AB; PDGF-BB, platelet-derived growth factor-BB; BMP-2, bone morphogenetic protein 2; FGF-2, fibroblast growth factor 2; VEGF, vascular endothelial growth factor.

A cumulative analysis for all donors at all time points revealed that PDGF-BB was the growth factor present at the highest concentration in liquid fibrinogen (56.567,5 pg/mL), whereas BMP-2, FGF-2, and VEGF showed the lowest concentrations (3.137,05 pg/mL, 2.636,31 pg/mL, and 2.458,94 pg/mL, respectively) (Figure 3A–C).

### 3.3. Correlation Analysis between Growth Factor Release and Platelet Accumulation

**Figure 4:**
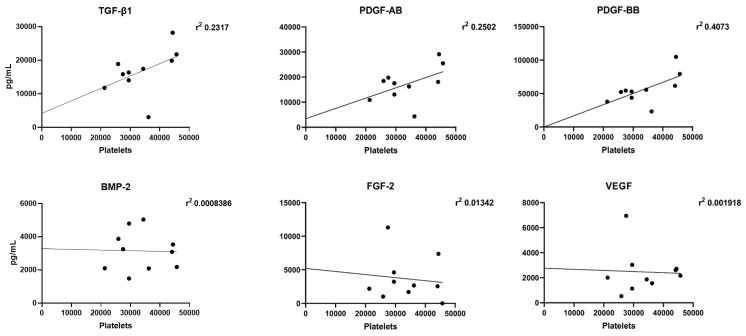
Correlation between released growth factors and accumulation of platelets. Statistical analyses were performed to correlate the release of growth factors and the platelet accumulation.

In order to verify the presence of a correlation between growth factor release and platelet count, a statistical analysis for each donor was performed. TGF-β1, PDGF-AB, and PDGF-BB levels correlated to platelet accumulation (*r*^2^ = 0.2317, *r*^2^ = 0.2502, and *r*^2^ = 0.4073, respectively), whereas, no correlation between BMP-2, FGF-2, and VEGF levels and platelet accumulation was found (*r*^2^ = 0.0008, *r*^2^ = 0.0139, and *r*^2^ = 0.0019, respectively) (Figure 4).

## 4. Discussion

In the last few years, there has been increased interest in possible methods to improve wound healing, particularly in surgical disciplines, including oral and maxillofacial surgery [6,7,8,9,10].

Previous studies showed that fibrin clots, such as L-PRF, are able to release a large amount of growth factors over time from alpha granules of platelets [20,21,22,23]. In this in vitro study, the release of growth factors, measured at three time points, and the correlation between growth factor release and cell accumulation were analyzed.

The subjects included in the study were volunteers aged between 40 and 50 years; this range corresponds to the age when people commonly undergo oral and periodontal surgery but when they are young enough not to have diseases typical of elderly people. Our aim was then to analyze the liquid fibrinogen from individuals representative of the average patient and, above all, were not administered with anticoagulant and antiplatelet medical therapy.

As the first analysis, the cell count test was performed and found a statistically significant mean accumulation of platelets of almost 1.5-fold in the liquid fibrinogen compared to whole blood samples. In contrast, no accumulation of all other blood cells was found in the liquid fibrinogen with a statistically significant decrease of lymphocytes and monocytes. These results partially confirm the findings of Varela et al., who reported a higher concentration of both platelets and lymphocytes in the liquid fibrinogen compared to the whole blood [24]. A possible explanation for these contrasting results could be the difference in centrifugation parameters (700 rpm in the work by Varela et al. vs. 2700 rpm of our protocol) and the collection point (Varela et al. collected the liquid fibrinogen as closely as possible to the red blood cells). Interestingly, the amount of neutrophils in the liquid fibrinogen obtained by our centrifugation device was around 1.5% of the amount of neutrophils present in whole blood samples. It is well-known that the presence of activated neutrophils affects the properties and the lifespan of fibrin clots [25]. Growth factors and cytokines delivery can rely on various physical properties of the fibrin scaffold such as entrapment of proteins within the scaffold and/or protein binding to the scaffold. Activated neutrophils mediate the fibrin degradation by producing serine proteases, and these latter enzymes can also affect the denaturation rate of cytokines present in the liquid fibrinogen. Consequently, neutrophils can influence the release of cytokines and growth factors from the platelet concentrates. Accordingly, the overall leukocyte amount can play an important role in determining satisfactory regenerative outcomes. Our results agree with the findings of Miron et al. [26] who observed low amounts of leukocytes in the liquid fibrinogen obtained with the same protocol. That work also has the merit of demonstrating that the liquid fibrinogen (liquid-PRF) obtained by centrifugation is not homogeneous in terms of cell concentration, highlighting that both platelets and leukocytes are mainly located at the interface between the yellow and red phase. This means that different experimental results may depend on the precise layer that is collected and that this information is worthy of inclusion in articles dealing with this topic.

Then, we analyzed the release over time of TGF-β1, PDGF-AB, PDGF-BB, BMP-2, FGF-2, and VEGF in liquid fibrinogen from each donor up to 14 days after collection. TGF-β1 is a secreted protein that regulates many cellular functions, including the control of immune and stem cell growth, proliferation, differentiation, and apoptosis [27,28]. Thus, the release of this factor is desirable in wound healing sites and particularly in the oral cavity where several types of cells, as fibroblasts and osteoblasts, have to be stimulated to proliferate. In our liquid fibrinogen, the TGF-β was stimulated mainly 7 days after collection, even if the release values at T0 and T14 were sufficiently high. PDGF plays a significant role in blood vessel production (angiogenesis) and regulates the proliferation of stem cells that can differentiate into fibroblasts, osteoblasts, and vascular smooth muscle cells [29]. We analyzed the presence and amount of two isoforms of PDGF, finding that PDGF-AB increased 7 days after collection, remaining at the same level at T14. Very interestingly, PDGF-BB was released at very high levels at T0, remained at the same level at T7, and further increased at T14, suggesting that this factor is primarily responsible for the fast healing of oral wounds, in accordance with what was demonstrated by Evrova et al. in tendon healing [30].

A cumulative analysis for all donors at all time points revealed that PDGF-BB was the growth factor present at the highest concentration in the liquid fibrinogen, unlike another study in which TGF-β1 was the most released [31]. It has to be underlined, however, that those authors did not analyze the secretion of PDGF-BB, as we did. In our experimental conditions, after PDGF-BB, the most expressed factor was TGF-β, in accordance with Castro et al. [31].

We also analyzed the release of VEGF, FGF-2, and BMP-2. VEGF is a growth factor that stimulates the formation of blood vessels. In particular, it is involved in both vasculogenesis (de novo formation of blood vessels) and angiogenesis (blood vessels growth from pre-existing vasculature) [32]. FGF-2, also known as basic Fibroblast Growth Factor (bFGF), is involved in angiogenesis, wound healing, and stimulates the proliferation and differentiation of pre-osteoblasts and fibroblasts [33]. BMP-2 plays an important role in the development of bone and cartilage. Like other proteins from the BMP family, BMP-2 has been demonstrated to induce osteoblast differentiation [34]. All these three growth factors were released at very low levels. Moreover, no correlation was found between the amount of these factors and platelet accumulation, suggesting that these factors are released by lymphomononuclear cells, which are not accumulated in the liquid fibrinogen.

VEGF and FGF-2 increased 7 days after collection and decreased after 14 days even if the level remained higher than levels measured at T0. The major part of the analyzed samples showed an increase of BMP-2 at 7 days after collection and the increase was still evident after 14 days. BMP-2 is an important factor in facture repair and it has a pivotal role in the initiation of repair processes [35]. In a previous study by Kalén et al., it was found that BMP-2 is released by platelets mainly at low pH [36], as in inflammation sites. Thus, considering that we measured the BMP-2 release in physiological conditions, and that we left the samples on the bench to analyze the release of factors at T0, T7, and T14 without adding any substances, apart from EDTA, this could explain the low production of BMP-2 observed in our study. However, since the development of an acidic tissue environment is common in wound healing sites [37], the use of the liquid fibrinogen, for instance at implant sites, may enhance the repair processes as the low pH contributes to stimulate the release of BMP-2. During bone healing, the pH becomes neutral and finally alkaline, thus stimulating alkaline phosphatase and osteocalcin [36].

In the future, novel platelet concentrates may be based on a better knowledge of growth factor kinetics and novel centrifugation modalities, such as horizontal centrifugation systems [26], to obtain higher cell concentrations. Until that time, more studies are warranted to validate these findings.

## 5. Conclusions

To the authors’ knowledge, no study has investigated the release kinetics of six different growth factors in liquid fibrinogen and their correlation with platelets. The results obtained from our study have shown that the liquid fibrinogen obtained by blood centrifugation does contain concentrated platelets and important growth factors for tissue repair and regeneration. The liquid fibrinogen obtained with the described method is particularly interesting both for the release of very high amount of PDGFs and for the almost complete absence of neutrophils. Among the growth factors analyzed, TGF-β1, PDGF-AB, and PDGF-BB were also found to be directly correlated with platelet accumulation. Finally, though without an univocal trend, the peak of release of the growth factors seems to be reached at around 7 days.

## References

[1] Miron R., Zucchelli G., Pikos M.A., Salama M., Lee S., Guillemette V., Fujioka-Kobayashi M., Bishara M., Zhang Y., Wang H.-L. (2017). Use of platelet-rich fibrin in regenerative dentistry: A systematic review. Clin. Oral Investig..

[2] Marx R.E., Carlson E.R., Eichstaedt R.M., Schimmele S.R., Strauss J.E., Georgeff K.R. (1998). Platelet-rich plasma: Growth factor enhancement for bone grafts. Oral Surg. Oral Med. Oral Pathol. Oral Radiol. Surg. Endodontol..

[3] Whitman D.H., Berry R.L., Green D.M. (1997). Platelet gel: An autologous alternative to fibrin glue with applications in oral and maxillofacial surgery. J. Oral Maxillofac. Surg..

[4] Bielecki T., Ehrenfest D.M.D. (2012). Platelet-rich plasma (PRP) and Platelet-Rich Fibrin (PRF): Surgical adjuvants, preparations for in situ regenerative medicine and tools for tissue engineering. Curr. Pharm. Biotechnol..

[5] Simonpieri A., Del Corso M., Vervelle A., Jimbo R., Inchingolo F., Sammartino G., Ehrenfest D.M.D. (2012). Current knowledge and perspectives for the use of platelet-rich plasma (PRP) and platelet-rich fibrin (PRF) in oral and maxillofacial surgery part 2: Bone graft, implant and reconstructive surgery. Curr. Pharm. Biotechnol..

[6] Bielecki T., Ehrenfest D.M.D., Everts P.A., Wiczkowski A. (2012). The role of leukocytes from L-PRP/L-PRF in wound healing and immune defense: New perspectives. Curr. Pharm. Biotechnol..

[7] Cieslik-Bielecka A., Choukroun J., Odin G., Ehrenfest D.M.D. (2012). L-PRP/L-PRF in esthetic plastic surgery, regenerative medicine of the skin and chronic wounds. Curr. Pharm. Biotechnol..

[8] Zumstein M.A., Rumian A., Lesbats V., Schaer M., Boileau P. (2014). Increased vascularization during early healing after biologic augmentation in repair of chronic rotator cuff tears using autologous leukocyte- and platelet-rich fibrin (L-PRF): A prospective randomized controlled pilot trial. J. Shoulder Elb. Surg..

[9] Dohan Ehrenfest D.M., Bielecki T., Jimbo R., Barbé G., Del Corso M., Inchingolo F., Sammartino G. (2012). Do the fibrin architecture and leukocyte content influence the growth factor release of platelet concentrates? An evidence-based ansie comparing a pure platelet-rich plasma (P-PRP) gel and a leukocyte- and platelet-rich fibrin (L-PRF). Curr. Pharm. Biotechnol..

[10] Del Corso M., Vervelle A., Simonpieri A., Jimbo R., Inchingolo F., Sammartino G., Ehrenfest D.M.D. (2012). Current knowledge and perspectives for the use of platelet-rich plasma (PRP) and platelet-rich fibrin (PRF) in oral and maxillofacial surgery part 1: Periodontal and dentoalveolar surgery. Curr. Pharm. Biotechnol..

[11] Del Fabbro M., Bortolin M., Taschieri S. (2011). Is autologous platelet concentrate beneficial for post-extraction socket healing? A systematic review. Int. J. Oral Maxillofac. Surg..

[12] Ehrenfest D.M.D., Rasmusson L., Albrektsson T. (2009). Classification of platelet concentrates: From pure platelet-rich plasma (P-PRP) to leucocyte- and platelet-rich fibrin (L-PRF). Trends Biotechnol..

[13] Ehrenfest D.M.D., Andia I., Zumstein M.A., Zhang C.-Q., Pinto N.R., Bielecki T. (2014). Classification of platelet concentrates (Platelet-Rich Plasma-PRP, Platelet-Rich Fibrin-PRF) for topical and infiltrative use in orthopedic and sports medicine: Current consensus, clinical implications and perspectives. Muscle Ligaments Tendons J..

[14] Ehrenfest D.M.D. (2010). How to optimize the preparation of leukocyte- and platelet-rich fibrin (L-PRF, Choukroun’s technique) clots and membranes: Introducing the PRF Box. Oral Surg. Oral Med. Oral Pathol. Oral Radiol. Surg. Endodontol..

[15] Dohan Ehrenfest D.M., de Peppo G.M., Doglioli P., Sammartino G. (2009). Slow release of growth factors and thrombospondin-1 in Choukroun’s platelet-rich fibrin (PRF): A gold standard to achieve for all surgical platelet concentrates technologies. Growth Factors.

[16] Pankov R. (2002). Fibronectin at a glance. J. Cell Sci..

[17] Mourão C.F., Valiense H., Melo E.R., Maia M.D.-C. (2015). Obtention of injectable platelets rich-fibrin (i-PRF) and its polymerization with bone graft: Technical note. Revista do Colégio Brasileiro de Cirurgiões.

[18] Lollobrigida M., Maritato M., Bozzuto G., Formisano G., Molinari A., De Biase A. (2018). Biomimetic Implant Surface Functionalization with Liquid L-PRF Products: In Vitro Study. BioMed Res. Int..

[19] Miron R., Pinto N.R., Quirynen M., Ghanaati S. (2019). Standardization of relative centrifugal forces in studies related to platelet-rich fibrin. J. Periodontol..

[20] Miron R., Kandalam U., Choukroun J., Fujioka-Kobayashi M., Hernandez M., Zhang Y., Ghanaati S. (2017). Injectable platelet rich fibrin (i-PRF): Opportunities in regenerative dentistry?. Clin. Oral Investig..

[21] Passaretti F., Tia M., D’Esposito V., De Pascale M., Del Corso M., Sepulveres R., Liguoro D., Valentino R., Beguinot F., Formisano P. (2013). Growth-promoting action and growth factor release by different platelet derivatives. Platelets.

[22] Schär M.O., Diaz-Romero J., Kohl S., Zumstein M.A., Nesic D. (2015). Platelet-rich Concentrates Differentially Release Growth Factors and Induce Cell Migration In Vitro. Clin. Orthop. Relat. Res..

[23] Wang X., Zhang Y., Choukroun J., Ghanaati S., Miron R. (2017). Behavior of Gingival Fibroblasts on Titanium Implant Surfaces in Combination with either Injectable-PRF or PRP. Int. J. Mol. Sci..

[24] Varela H.A., Souza J., Nascimento R.M., Araújo R.F., Vasconcelos R.C., Cavalcante R.S., Guedes P.M., Araújo A.A. (2018). Injectable platelet rich fibrin: Cell content, morphological, and protein characterization. Clin. Oral Investig..

[25] Kruger P., Saffarzadeh M., Weber A.N.R., Rieber N., Radsak M., Von Bernuth H., Benarafa C., Roos D., Skokowa J., Hartl D. (2015). Neutrophils: Between Host Defence, Immune Modulation, and Tissue Injury. PLoS Pathog..

[26] Miron R.J., Chai J., Zheng S., Feng M., Sculean A., Zhang Y. (2019). A novel method for evaluating and quantifying cell types in platelet rich fibrin and an introduction to horizontal centrifugation. J. Biomed. Mater. Res. Part A.

[27] Massagué J., Xi Q. (2012). TGF-β control of stem cell differentiation genes. FEBS Lett..

[28] Li M.O., Flavell R.A. (2008). TGF-β: A Master of All T Cell Trades. Cell.

[29] Fredriksson L., Li H., Eriksson U. (2004). The PDGF family: Four gene products form five dimeric isoforms. Cytokine Growth Factor Rev..

[30] Evrova O., Buschmann J. (2017). In vitro and in vivo effects of PDGF-BB delivery strategies on tendon healing: A review. ECM.

[31] Castro A., Cortellini S., Temmerman A., Li X., Pinto N., Teughels W., Quirynen M. (2019). Characterization of the Leukocyte- and Platelet-Rich Fibrin Block: Release of Growth Factors, Cellular Content, and Structure. Int. J. Oral Maxillofac. Implant..

[32] Ferrara N., Houck K., Jakeman L., Leung D.W. (1992). Molecular and biological properties of the vascular endothelial growth factor family of proteins. Endocr. Rev..

[33] Okada-Ban M., Thiery J.P., Jouanneau J. (2000). Fibroblast growth factor-2. Int. J. Biochem. Cell Biol..

[34] Xue T., Wei L., Qiao L., Qiu J., Zha D. (2009). Does bone morphogenetic proteins play an important role in chronic rhinosinusitis?. Med. Hypotheses.

[35] Tsuji K., Bandyopadhyay A., Harfe B.D., Cox K., Kakar S., Gerstenfeld L., Einhorn T., Tabin C.J., Rosen V. (2006). BMP2 activity, although dispensable for bone formation, is required for the initiation of fracture healing. Nat. Genet..

[36] Kalén A., Wahlström O., Linder C.H., Magnusson P. (2008). The content of bone morphogenetic proteins in platelets varies greatly between different platelet donors. Biochem. Biophys. Res. Commun..

[37] Zhang Z., Lai Q., Li Y., Xu C., Tang X., Ci J., Sun S., Xu B., Li Y. (2017). Acidic pH environment induces autophagy in osteoblasts. Sci. Rep..

